# Early warning signals detect critical impacts of experimental warming

**DOI:** 10.1002/ece3.2339

**Published:** 2016-07-29

**Authors:** Lauren Jarvis, Kevin McCann, Tyler Tunney, Gabriel Gellner, John M. Fryxell

**Affiliations:** ^1^ Department of Integrative BiologyUniversity of GuelphGuelphOntarioN1G 2W1Canada; ^2^ Center for LimnologyUniversity of Wisconsin MadisonMadisonWisconsin53706; ^3^ Department of Environmental Science and PolicyUniversity of California DavisDavisCalifornia95616

**Keywords:** Climate warming, early warning signals, ecology, population extinction, statistical indicators of collapse, thermal performance

## Abstract

Earth's surface temperatures are projected to increase by ~1–4°C over the next century, threatening the future of global biodiversity and ecosystem stability. While this has fueled major progress in the field of physiological trait responses to warming, it is currently unclear whether routine population monitoring data can be used to predict temperature‐induced population collapse. Here, we integrate trait performance theory with that of critical tipping points to test whether early warning signals can be reliably used to anticipate thermally induced extinction events. We find that a model parameterized by experimental growth rates exhibits critical slowing down in the vicinity of an experimentally tested critical threshold, suggesting that dynamical early warning signals may be useful in detecting the potentially precipitous onset of population collapse due to global climate change.

## Introduction

Climate warming exposes populations to novel environmental pressures that could potentially induce a broad suite of complex ecological responses (Walther et al. 2002; Thomas et al. 2004; Post 2013) leading to the collapse of one or more species. While it is clear that rising temperature has already played a major role in local extinction and shifts in species distribution (Thomas et al. 2004; Parmesan 2006), it is far less clear which species are most vulnerable to future impacts of global warming because thermal environmental conditions modify a broad suite of individual traits (e.g., metabolism, foraging rates, and growth rates) that collectively determine an organism's demographic response to temperature change within a complex network of interactions (Dell et al. 2014; Fussmann et al. 2014). The ability to forecast how thermal changes might impact species, and their interactions, is critical if we are to move toward the ultimate goal of understanding the cascading effects of climate warming on biodiversity and ecosystem function (Tunney et al. 2012; Post 2013).

Researchers have made substantial progress in cataloguing the responses of organismal traits to thermal changes (Huey and Stevensen 1979; Huey and Kingsolver 1989; Savage et al. 2004; Dell et al. 2011). These trait responses, measured across thermal gradients, have been coined thermal performance curves, describing individual performance as a function of changing environmental conditions (Huey and Stevensen 1979; Kingsolver and Gomulkiewicz 2003). Synthesizing the set of all thermal performance curves (e.g., metabolism, growth, foraging etc.) for a given species provides the first steps toward an integrated climate theory with the ability to predict climate influences on individual populations to whole ecosystems (Gilbert et al. 2014). Although not yet considered, such empirical cataloguing of thermal performance curves may also allow us to unite climate change literature with recent research on the development of early warning signals – a body of work where ecologists seek to identify informative statistical indicators that precede species, or ecosystem, collapses that are driven by gradual environmental deterioration (Drake and Griffen 2010; Veraart et al. 2012).

**Figure 1:**
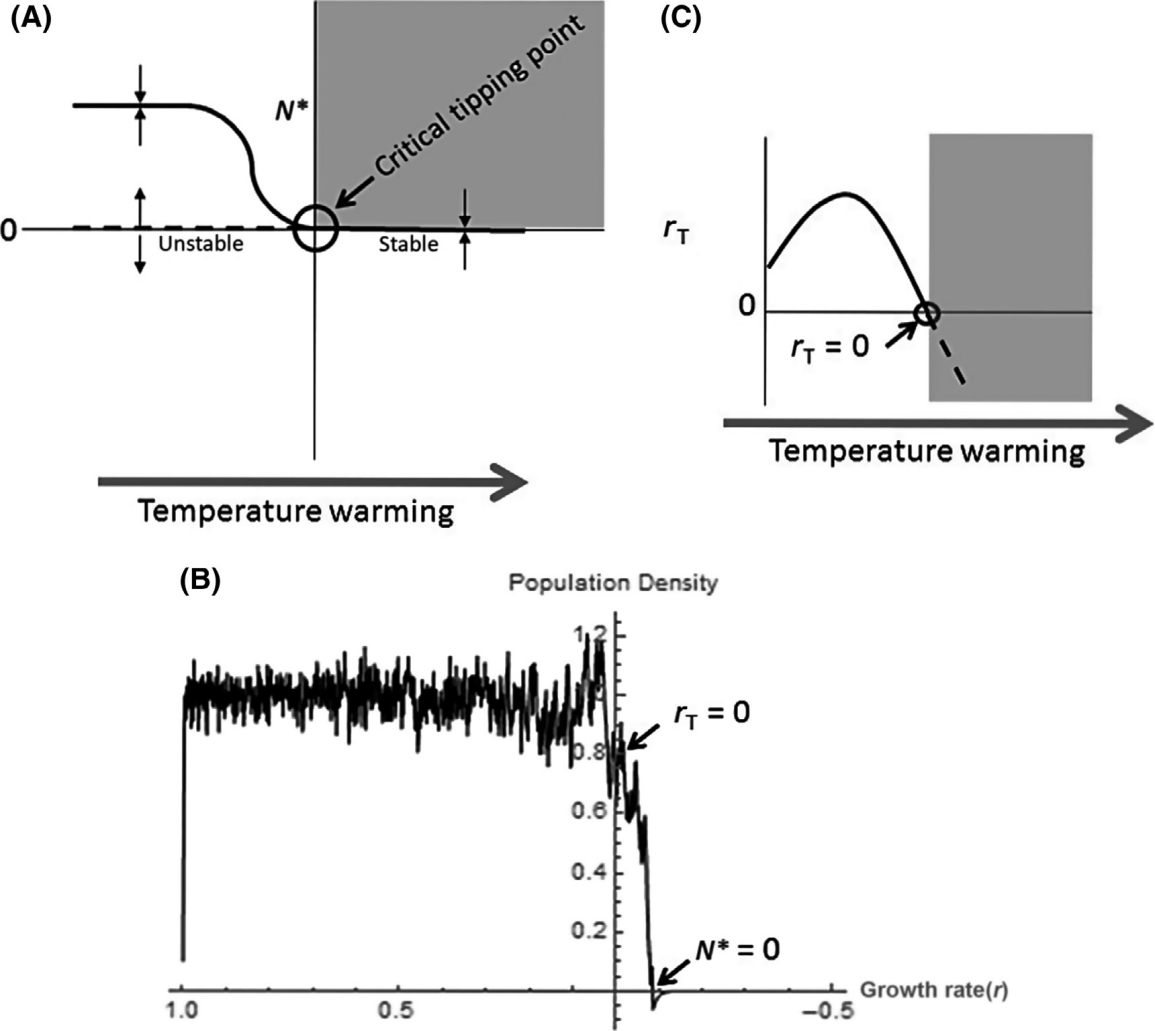
Critical tipping point and example thermal performance curves. Hypothetical depiction of a critical tipping point and thermal performance curves with temperature warming. (A) The critical tipping point, or transcritical bifurcation, exists where the stabilities of two different fixed points switch, such that a stable equilibrium (N*) of a positive density is replaced by a stable equilibrium of zero density. Arrows indicate the direction of nearby trajectories, where they are attracted to stable (solid lines) equilibria and repelled by unstable (dashed lines) equilibria. Here, as demonstrated in plot (B) the critical tipping point is reached when *r*_T_ < 0, which is determined by the temperature sensitivity of this key parameter, *r* (C) (but note that a critical tipping point can occur with any change in conditions that affects underlying parameters). When *r*_*T*_ ≤* *0, the population cannot recover from a perturbation, and beyond this point, the carrying capacity is undefined and extinction is inevitable. The gray boxes in (A) and (C) indicate the conditions – after a threshold has been breached – where populations can no longer persist.

Early warning signals use a common dynamical property of equilibrium systems known as critical slowing down (Boettiger and Hastings 2013), whereby a system experiences a weak compensatory recovery toward equilibrium following perturbation. This characteristic is often indicative of an approach toward a local bifurcation, or critical tipping point that represents the boundary zone between population persistence and extinction (Fig. 1) (Wissel 1984; Hugget 2005; Scheffer et al. 2009; Drake and Griffen 2010; Veraart et al. 2012; Boettiger and Hastings 2013). Critical slowing down is accompanied by other related statistical signatures of an impending species loss or ecosystem collapse, namely increased degree of temporal autocorrelation and increasing variance in population densities over time (Dakos et al. 2012; Krkosek and Drake 2014). Delayed recovery yields greater similarities in population abundance between adjacent time steps (increased autocorrelation) and additive disturbance effects that may cause the population to deviate further from mean densities following perturbation (i.e., variance increases if the population is disturbed multiple times before it has recovered to its preperturbation state). Although the early warning signal approach has become more widespread (Drake and Griffen 2010; Carpenter et al. 2011; Veraart et al. 2012), it has not yet been used for predicting climate‐induced tipping points in populations, which one would predict due to the temperature dependence of key population traits and the perilous state of global warming (Brown et al. 2004; Englund et al. 2011).

As a first step toward the long‐term goal of uniting thermal performance curves with ecological theory and predicting population dynamics and species loss, here we explore the simplest experimental scenario of a producer population (algae; *Chlorella vulgaris*) growing on a limiting nutrient and induce a transcritical bifurcation (i.e., a bifurcation that has some special “nonlinearities” at the point of bifurcation as opposed to the generic form, which is effectively two lines of equilibria crossing and exchanging stability properties). Our approach is to first derive thermal performance curves empirically for growth rate (*r*) and carrying capacity (*K*), two easily measurable biological parameters that match those of classical single‐species models. We then apply these parameter responses to a discrete time logistic growth model to predict how algal population dynamics should change across a relevant gradient in temperature. These predictions are then compared to five replicated experiments spanning a range of temperature conditions to explicitly test (1) whether climate warming leads to algal extinction and (2) whether populations on the verge of extinction displayed the early warning signals predicted by the theoretical model. Specifically, we compare theoretical patterns of autocorrelation, recovery rates, and coefficient of variation with those of the experimental population replicates. This procedure allows us to document the existence of early warning signals in response to climate warming that are completely consistent with well‐established thermal performance curves of key organismal traits, suggesting exciting new potential for the prediction of temperature‐induced tipping points of impending collapse.

## Materials and Methods

We estimated the thermal performance curves for *r* and *K* from the time series data of a simple quasi‐chemostat algae‐nutrient experimental system exposed to temperatures of 15, 20, 25, 30, and 35°C (eqs. (2) and (3), respectively). These thermal performance curves were used to parameterize a Ricker logistic population growth model in discrete time (Ricker 1954), making the model temperature dependent (eq. 1a). We felt the Ricker model in discrete time nicely matched the simplicity of our experiment. It reasonably mimics single‐species microcosm experiments (chemostat models regularly yield logistic dynamics, Kooi et al. 1998), yields a range of bifurcations and importantly, like almost all biologically motivated dynamic models readily begets a transcritical. Thus, the Ricker model always has a trivial equilibrium at N* = 0. This model constraint, biologically driven, thus makes the transcritical bifurcation generic (i.e., the interior equilibrium collides and passes through the trivial equilibrium, which remains and drives a transcritical bifurcation).

The model‐generated time series were perturbed every seventh time step (eq. 1b) and then analyzed to estimate recovery rates, autocorrelation structure, and the coefficient of variation to make predictions about the behavior of these statistical indicators in the proximity of a critical tipping point. Model predictions were then compared to the observed patterns of autocorrelation, return time, and coefficient of variation of algal population densities analyzed from the experimental time series data.

### The experiment

#### Setup

**Figure 2:**
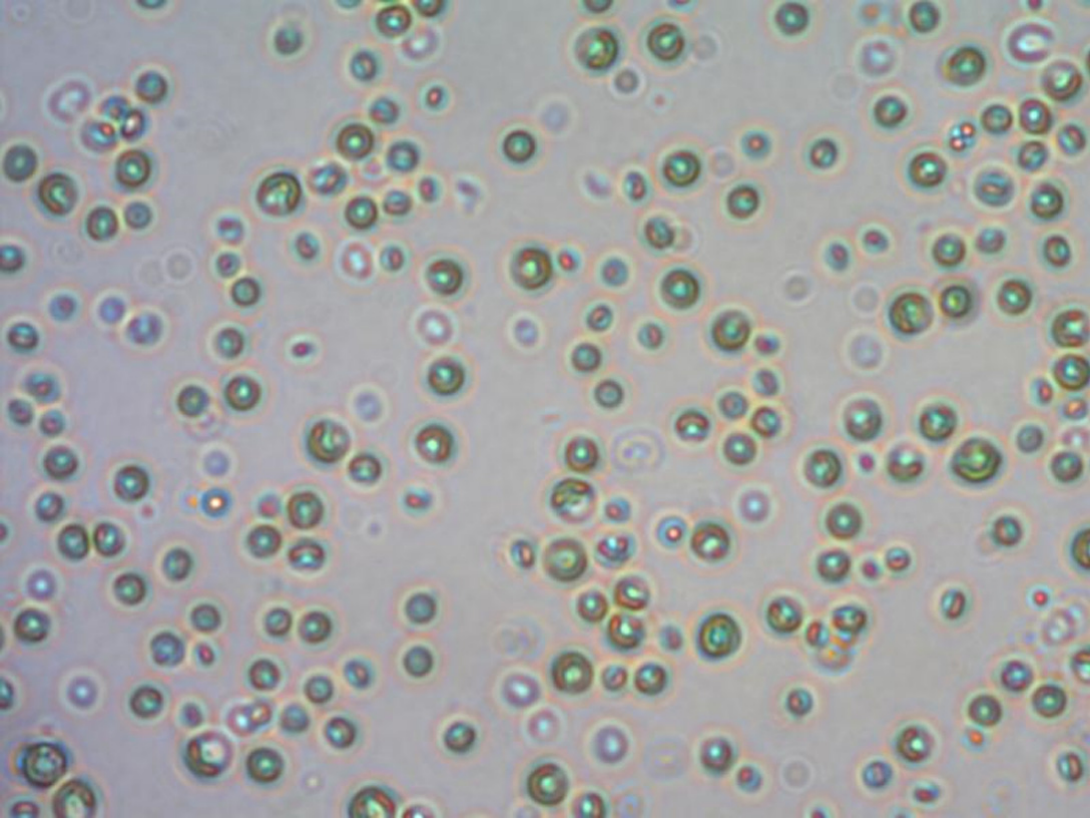
*Chlorella vulgaris*. This is a photograph of our experimental organism under 20**×** magnification using a compound microscope.

*Chlorella vulgaris* is a freshwater species of unicellular green algae that is common in North America (Beyerinck 1890) (Fig. 2). A stock population of *C. vulgaris* (strain #90) was obtained from the Canadian Phycological Culture Centre (CPCC) in Waterloo, Ontario. This culture was maintained at the University of Guelph, in COMBO growth medium (Kilham et al. 1998) at 20°C in an incubator (Fisher Isotemp® Low Temperature Incubator Model 307C). All subsequent algal growth experiments were conducted in a closed chamber at the Hagen Aqualab at the University of Guelph lit with fluorescent lights (955 lux, measured at a uniform distance across containers) operating on a 12:12 light/dark cycle, and consisting of 15 replicate algae cultures grown in 150‐mL flasks. Prior to temperature trials, each flask was inoculated with 125 mL of algae from the common batch culture, yielding starting densities (*t* = 0) of ~4.65 × 10^6^ cells/mL^3^ (Rip et al. 2010). Each flask was then randomly assigned to one of five temperature treatments (three replicates per treatment) consisting of water baths (five 45‐L coolers) that were held at a constant temperature of either 15, 20, 25, 30, or 35°C (±1°C) using thermostat‐regulated heaters. These temperatures were chosen to represent optimal and suboptimal temperatures for algal growth (Ahlgren 1987), as well as annual temperature variation that recognizes an expected 1–7°C increase with global warming in freshwater lakes (Ficke et al. 2007).

#### Experimental population estimates

Time series data were obtained by measuring the fluorescence of 3 mL of algal samples every 1–4 days for the 64‐day experimental period. Samples were mixed thoroughly, withdrawn from flasks selected in random order and then returned to the flask to avoid significant changes in volume by the end of the study. Fluorescence data (e.g., values of 620, 750, 380, 220, 2.4 for temperatures 15, 20, 25, 30, and 35°C, respectively) were normally distributed and were regressed against algal cell counts (obtained using a hemocytometer in preliminary experiments), allowing us to convert fluorescence to cell density for subsequent time series analyses (*y* = 11985*x* + 261290; *R* ^2^ = 0.925504). Using the program ImageJ1.42 (NIH), images of algal cells on the hemocytometer were used to measure cell diameter, noting that cell volume did not vary significantly with temperature over the duration of preliminary experiments.

#### Adding experimental perturbations

*Chlorella vulgaris* populations were perturbed to observe how a population responds to a disturbance under increasing thermal pressure, and thus, how certain statistical indicators (recovery rates, autocorrelation, and coefficient of variation) change in the vicinity of a temperature‐driven critical tipping point. Growth rate is a property of the population that is only seen when the population is pulled away from the equilibria. By adding targeted perturbations, we were able to compare more uniform rates of increase across temperature treatments, and calculate growth estimates from a wider range of *N*_t_. In total, there were nine perturbation events that took place on day 8, 15, 21, 29, 37, 43, 49, and 55 (every 4–7 days). These events were chosen based on balancing the time required for the populations growth rates to saturate and avoiding nutrient depletion. Preliminary work suggested this range was reasonable. Experimental populations were perturbed by replacing 50 mL of algae in solution (~40% of the population density) from each culture with COMBO made with 50% of its prescribed nutrient content (i.e., only 0.5 mL/L of K_2_HPO_4_, NaNO_3,_ and Na_2_EDTA2H_2_O instead of 1.0 mL/L) (Kilham et al. 1998). Using the prescribed nutrient concentration resulted in algal growth that yielded densities beyond that which could be accurately measured by our fluorometer and hemocytometer.

#### Uniform and stationary data

To perform time series analyses, some basic assumptions must be met. First, time series data are required to be uniform, such that successive data points are equally spaced in time. We analyzed our data with missing observations and interpolated data, but we present statistics without interpolation to avoid the potential influence of autocorrelation coefficients. Second, the time series must be stationary, where its statistical properties remain constant over a given time. This is crucial to ensure that the patterns in variance and autocorrelation tested here are signatures of critical slowing down and not an effect of time (or any other external process) (Chatfield 2014). Although our populations were disturbed, our methods ensured to our best ability that there were no underlying processes driving nonstationary dynamics. We performed analyses on the raw data for all temperatures. A linear, quadratic, and null model were compared for all temperature treatments to test for stationarity.

### The model

Theoretical population predictions were generated using the Ricker logistic growth model

(1a) $$N_{t+1}=N_{t}e^{r_{T}\left(1-\;\frac{N_{t}}{K_{T}}\right)}+p$$

where *N*_*t*_ is the population size at time *t*,* N*_*t+1*_ is the population size at the next time step, *r*_*T*_ is the temperature‐dependent growth rate, *K*_*T*_ is the temperature‐dependent carrying capacity, and *p* is the perturbation given every seventh time step (*t*) (4‐ to 7‐day time steps produced similar results).

The model was perturbed by removing a constant portion of the algal population before restoring to a constant volume;

(1b) $$p=\left\{\begin{array}{cc} -0.4N_{t} & \;\text{if tmod}\;7=0\\ 0 & \text{otherwise} \end{array}\right.$$

After each perturbation, the population's ability to recover is determined by the growth term, $r_{T}\left(1-N_{t}/K_{T}\right)$, where *r*_*T*_ and *K*_*T*_ are the temperature‐dependent maximum exponential rate of increase (*r*) and carrying capacity (*K*), respectively. For any given population, the growth term determines its dynamical characteristics; *N*_*t*_ approaches *K*_*T*_ at a rate, *r*_*T*_, such that at relatively low values of *r*_*T*_ (*r*_*T*_ *< N*_*t*_ */K*_*T*_) the entire growth term will approach zero (i.e., growth of the population slows as the carrying capacity is reached) and at relatively high values of *r*_*T*_ (*r*_*T*_ *> N*_*t*_ */K*_*T*_) the population will exceed its carrying capacity (i.e., overshoot) and the growth term becomes negative. Therefore, population size decreases (*N*_*t*_ >* N*_*t + 1*_) over time if *N*_*t*_ *> K*_*T*_ *,* increases over time if *N*_*t*_ *< K*_*T,*_ and stays the same when *N*_*t*_ *= K*_*T*_. For the latter, there is no change in population size from one time step to the next (*N*_*t + 1*_ *= N*_*t*_), and the system has reached equilibrium (N*).

### Temperature dependence of intrinsic rate of increase and carrying capacity estimated from an experimental population

#### Estimating r and K

**Figure 3:**
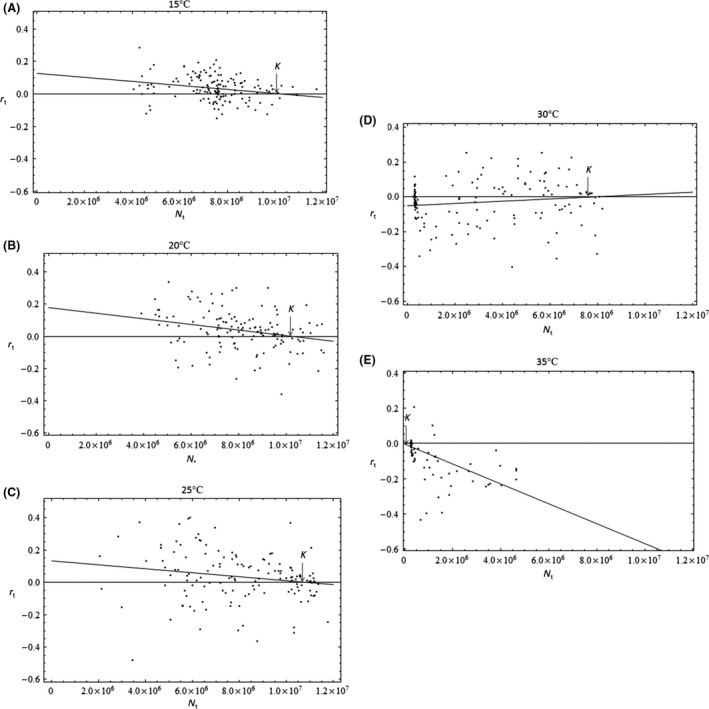
Linear regressions for estimating *r* and *K* from experimental data. Here, we show the linear models fit to exponential rate of increase (*r*_*t*_) and algal cell density (*N*_*t*_) data for each temperature treatment (15°C (A), 20°C (B), 25°C (C), 30°C (D), 35°C (E)). It should be noted that the *r* and *K* for 30°C and 35°C treatments cannot be accurately estimated from these regressions because it can be argued that these temperatures are beyond the thermal tolerance of the algae populations, in which case population growth rate is no longer limited by density, but rather physiological constraint. Only one of three regressions is displayed for each treatment temperature; however, the mean of three replicate *r* and *K* estimates were used to construct corresponding thermal performance curves. In the above plots, *r* is the y‐intercept and *K* is the *x*‐intercept.

Intrinsic rates of population increase (or instantaneous per capita growth rates), *r*_*t*,_ were calculated using the exponential growth rate equation, $r_{t}={\text{log}}_{e}\left(N_{t+1}/N_{t}\right)/\tau$, where *τ* is the number of days between consecutive density measurements (Fryxell et al. 2014). The exponential growth rate equation is convenient when the time between measurements is irregular, as was the case with our population data (Fryxell et al. 2014). *r*_*t*_ for each time series (each replicate) were estimated between consecutive measures of population density at a given time (*N*_*t*_). We then plotted *r*_*t*_ for each time interval against the corresponding *N*_*t*_ (density at the beginning of said interval) and used a linear model to describe the relationship of population growth with increasing abundance, assuming normal distribution (Fig. 3). It is assumed that maximum population growth (denoted here as *r*) occurs at densities close to zero; thus, we estimate *r* to be *r*_*t*_ when *N*_*t*_ *= *0. Further, the carrying capacity (*K*) exists at the maximum density that can be sustained by limiting resources, such as nutrients (the *x*‐intercept of the regression line for *r*_*t*_ vs. *N*_*t*_; *i.e*., *N*_*t*_ when *r*_*t*_ = 0).

It is important to note that there are three different scenarios in which temperature can affect *r* and *K* (see Fig. S1). Temperature variation can cause changes to either both parameters simultaneously (Fig. S1A) or to one parameter with no effect on the other (Fig. S1B and C). We found that *r* changed with rising temperatures, but that *K* was unaffected until thermal thresholds. Beyond threshold temperatures, estimates of *r* become negative, the populations experience extinction, and equilibrium exists at zero density. *K* could not be estimated confidently because actual nutrient levels were not measured, and thus, it remains unclear whether temperature affected the amount of available nutrients or the ability of *C. vulgaris* to utilize the nutrients (due to physiological limitations). Therefore, *K* was kept constant in the model time series analyses.

#### Estimating thermal performance curves

The thermal performance curve for *r* was determined by separately fitting curves through the five data points (mean of three replicates across five temperatures) and the curve with the lowest AIC score determined the fitted model that best represented the *r‐*temperature dataset (eq. (2)). *K* was kept at a constant value represented by the mean carrying capacity of the 15, 20 and 25°C treatments (eq. (3)) (selected models did not perform the null model; see Table S1 for AIC scores). The temperature dependencies of *r* and *K* (denoted as *r*_*T*_ and *K*_*T*_) were implemented in the population model (eq. 1a) to generate theoretical time series data using the parameter values that were estimated for each temperature treatment. In doing so, we produced theoretical time series data using experimentally determined parameter estimates.

(2) $$r_{T}=-0.847+0.1033T-0.003T^{2}$$

(3) $$K_{T}=6.016\times 10^{6}$$

### Analyzing early warning signals from a temperature‐parameterized model

We used three statistical indicators of critical slowing down as early warning signals of an approaching tipping point. We conducted simulation experiments by parameterizing a Ricker logistic growth population model with the experimentally derived thermal performance curves for *r* and *K* (see above subsection), and then examined the model dynamics of corresponding time series data.

#### Recovery rate

We estimated recovery rates – the return of a population to its preperturbation state – as the slope of ln(abundance) for each uninterrupted period between perturbation events (i.e., the population density immediately after one perturbation to the density just prior to the next perturbation). The mean recovery rate of the seven perturbations was calculated for each temperature.

#### Autocorrelation

Autocorrelation was analyzed across the entire time series for each replicate. Patterns in lag‐1 autocorrelation were measured using autoregressive coefficients of the autoregressive model (or process; AR(1)). This model is a simple linear difference equation (Chatfield 2014) (eq. (4)) whose coefficient, *ρ,* is calculated based on past density observations, *N*_*t,*_ and an estimate of the residual error, *ε*_*t*_ (for model analyses, *ε*_*t*_ has a mean of 0).

(4) $$N_{t+1}=\rho N_{t}+\varepsilon_{t}$$

For each temperature at which populations persisted, we normalized the model time series datasets by dividing by mean abundance and plotting *N*_*t*_ against *N*_*t*_ *+1*. The autocorrelation coefficient, *ρ,* is the slope of the fitted line.

#### Coefficient of variation

Lastly, the coefficient of variation in algal population density was determined using the entire algal time series to encompass all deviations from the mean population density in response to multiple perturbations under worsening conditions, using the formula CV=σ/μ , where *σ* is the standard deviation and *μ* is the mean population size.

### Early warning signals directly estimated for population time series

Experimental time series data were stationary (i.e., the distribution of the time series is not time‐dependent) and analyzed from days 8 to 56 (Chatfield 2014). Recovery rates, autocorrelation coefficients, and coefficient of variation in algal density were then estimated from the experimental time series, using the same metrics as earlier applied to the deterministic model.

## Results

**Figure 4:**
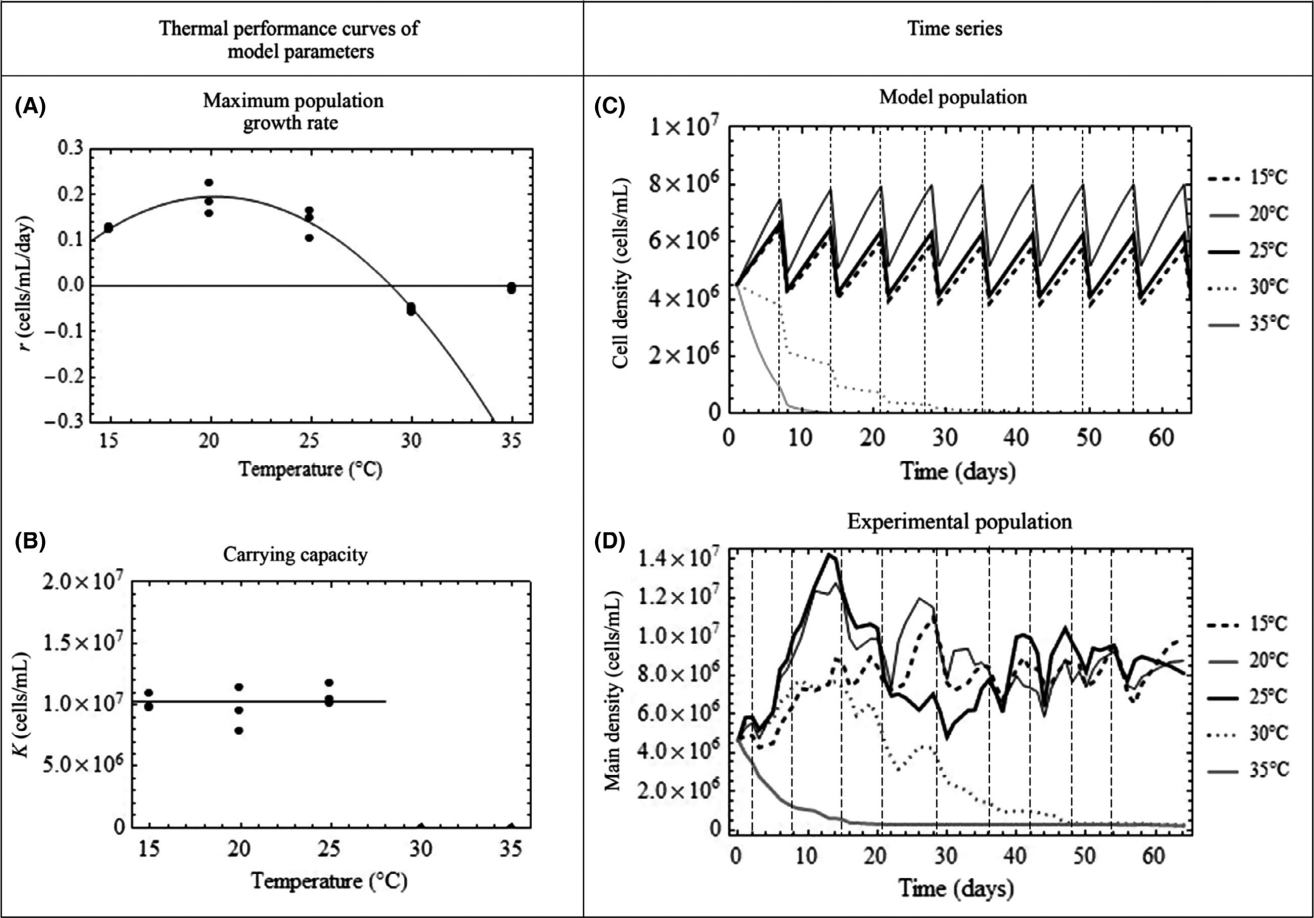
Thermal performance curves and time series data. Left panel: Thermal performance curves for (A) maximum intrinsic rate of increase, *r*, and (B) carrying capacity, *K*, at each temperature estimated from experimental time series data. Each point represents the estimated value of *r* and *K* from one replicate. Right panel: Time series data for the model (C) and experimental (D) populations. For the experimental time series, lines represent mean densities (cells mL^−1^) of three replicate cultures grown at each of the five temperature treatments (15, 20, 25, 30, 35°C) over the 64‐day experiment. Dashed vertical lines indicate perturbation events. Time series analyses were performed on data from days 8–56, or until a given replicate culture went extinct. Recovery rates were measured between vertical dotted lines, while and autocorrelation coefficients and coefficients of variation was measured across the entire time series.

Our experimentally derived thermal performance curve for the maximum exponential growth rate (*r*_*T*_) was unimodal in shape (Fig. 4A), such that *r* increased between 15°C and 20°C, peaked at 20°C, fell at temperatures exceeding 20°C, and crossed zero at 27°C; after which further warming resulted in negative growth rate estimates. On the other hand, our results showed that algal carrying capacity (*K*) showed no statistically detectable change (*P* > 0.05) (~8.0 × 10^6^ to 1.1 × 10^7^ cells/mL) across trials with a positive maximum rate of growth (15, 20 and 25°C; Fig. 4B). By definition, ecological carrying capacity is only meaningful in systems with positive *r*, so it was not possible to estimate effects on algal carrying capacity at higher environmental temperatures. The thermal performance curves accordingly suggest that the maximum rate of increase for *C. vulgaris* is temperature‐dependent, whereas carrying capacity is not.

Our central assumption is that increasing temperatures beyond thermal optima should push populations toward collapse. The temperature‐dependent Ricker model, based on thermal performance curves, predicts that *C. vulgaris* populations should be able to persist at temperatures up to 25°C, but collapse at 30°C and higher (Fig. 4C). In general, the model also predicts that algal populations should show increasingly faster recovery from perturbation at temperatures between 15 and 20°C, but increasingly slower recovery at higher temperatures. If populations do not return to preperturbation densities before the next disturbance, those that recover more quickly should reach higher densities between perturbation events, leading to fluctuations of greater amplitude. Beyond 25°C, increasingly warmer temperatures should lead to shorter times to extinction. Further, algal populations are predicted to be unsustainable at temperatures exceeding ~28°C (due to negative growth rates; Fig. 4A).

As predicted, experimental populations of *C. vulgaris* at the three lowest temperature treatments (15, 20, and 25°C) persisted for the duration of the experiment (Fig. 4D). The populations grown at 15°C appeared to be the most “well‐behaved,” displaying minor fluctuations in density, while algal populations subjected to the 20°C treatment demonstrated faster growth following perturbations, and thus slightly more variable dynamics. Consistent with this pattern, 25°C treatments persisted for the duration of the experiment, but were highly variable in density. Once perturbed, these populations reacted more slowly than algal cultures at lower temperatures, leading to delayed recovery to a preperturbation state. It should be noted that one of the three 25°C replicates started to rapidly decline in density at ~day 58. Similar to our simulated populations, experimental replicates subject to higher temperatures collapsed just before the conclusion of the experiment. Populations subjected to the 30°C treatment persisted until day 15, but inadequate recovery from perturbations resulted in population extinction by day 48. All experimental populations exposed to 35°C conditions experienced negative growth rates from the first day of experimentation until they reached extinction on day 18.

**Figure 5:**
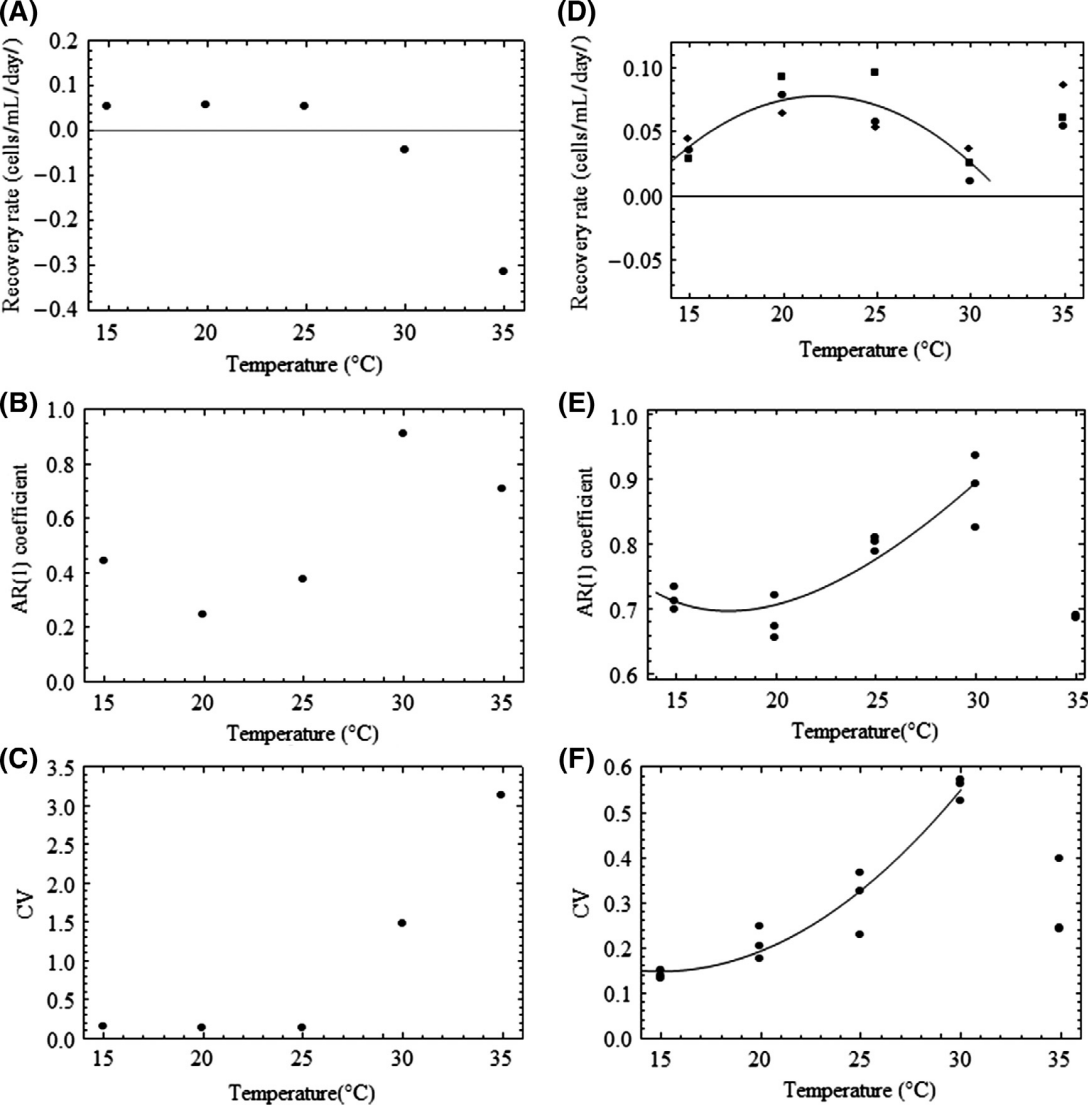
Early warning signals of a critical tipping point. Plots A, B and C represent model predictions for patterns in the recovery rate, lag‐1 autocorrelation (AR(1)) coefficient, and coefficient of variation. Plots D, E, and F represent corresponding patterns of the same statistical indicators for experimental observations. For plot D, each point represents the mean value of eight perturbation events for a given replicate. For plots E and F, each point is the AR(1) coefficient and coefficient of variation (respectively) for one replicate time series at a given temperature. Observed data for 30 and 35°C were not included in the curve fitting because they exceeded thermal tolerance. Best fit curves were selected using the Akaike information criterion (AIC). All best fit models outperformed the null model (slope = 0).

The simulated data suggest that early warning signals can be used to anticipate a thermally driven critical tipping point (Fig. 5); however, more data representing a wider spectrum of temperatures between optimal and critical points are required to evaluate the presence of CSD in an experimental population (Fig. 5D–F). Lag‐1 autocorrelation (AR(1)) coefficients of the model time series (Fig. 5B) were lowest around 20°C, where populations tended to recover more quickly from a perturbation. With warming beyond the apparent optimum temperature of 20°C (Fig. 4A), recovery rates decreased (Fig. 5A) and AR(1) coefficients increased as temperatures climbed toward 30°C. The concavity seen here alludes to a second, cold‐temperature threshold that was not tested in our study but further implies that CSD is a phenomenon that can be detected near temperature‐induced transitions on either end of the spectrum.

Finally, our model predictions of coefficient of variation were qualitatively similar to observed patterns near the thermal threshold (Fig. 5C and F). The coefficient of variation of our experimental population showed a more steady increase with warming, while our model predicted relatively constant variability in algal density, with higher coefficient of variation at 30°C. Hence, our model predictions were consistent with observations of AR(1) and recovery rates at ambient temperatures below 26°C, beyond which point subsequent increases in coefficient of variation with warming were more apparent. Recall that this was when we started to see population collapse, whereby populations deviate further from the mean and approach zero. While it is well known that increases in *r* can drive a period‐doubling cascade (experimentally determined parameters do not yield such a result), the results of our experiments come from the fact that the Ricker model also readily, like all population models, yields a transcritical bifurcation. It should also be noted that similar patterns in AR(1) coefficients and CV were observed when a stochasticity was added to our discrete model.

## Discussion

For the first time, we have observed the phenomenon of CSD due to temperature warming. Further, we document the existence of an early warning signal before a temperature‐induced critical transition in a model population parameterized by experimentally derived organismal traits, and we explicitly show the implications of warming on population function (i.e., stability; population persistence).

We have also reported unimodal patterns in growth rates across a temperature gradient, and a potentially discontinuous response in carrying capacity, a result that has been overlooked in previous climate studies yet emphasizes the asynchronous effect of temperature on biological rate responses (Savage et al. 2004; Dell et al. 2011). To further this point, we see extreme value in studying how the shape of certain thermal performance curves dictates when CSD may be detected, and thus, whether or not some traits might have more or less utility as early warning indicators. For example, the thermal performance curve of one trait might be more left‐shifted than another, and thus provide us with an earlier signal of an approaching transition point. Though beyond the scope of our study, it would also be intriguing to investigate the role of phenotypic plasticity in an organism's response to climate change and if subsequent changes to trait performance might potentially mask indicators of CSD before a thermally induced critical transition. Nonetheless, our work shows promise in mechanistically linking thermal performance theory with early warning signal theory to predict the onset of species extinction.

Growth rate and carrying capacity are potential parameters that could be used to indicate steady state transitions. In our study, slower growth rates mean higher risk of extinction; however, if we move up to more complex systems, faster growth at the consumer level may in fact lead to extinction due to stronger consumer–resource interactions (driving overshoot dynamics and oscillations that can collapse a population). Moving forward, studying early warning indicators will inevitably include the interplay of multiple performance curves in order to understand the general patterns that arise in a system exposed to external pressures (Englund et al. 2011; Fussmann et al. 2014). Like all early warning signal theory, it is about knowing your system's “normal” and being able to interpret deviations under various stressors in order to detect a transition. What we have shown here is how thermal performance theory closely maps to more common statistical indicators, and thus, the mechanistic underpinnings of critical slowing down with regard to the key biological rates associated with lost resilience. Further, in cases where it might be difficult to know certain vital rates (e.g., growth r) in more complex systems, other early warnings can still be reliably used.

One benefit of statistical early warning signatures in time series is the opportunity to detect an oncoming transition in an ecological system without knowledge of the specific variables driving the collapse. If a goal of climate research is to ameliorate the impacts of warming on species loss, however, then it may be important to be able to differentiate between temperature effects near critical points rather than those of other environmental variables. We have shown that early warning indicators, such as increased variability, autocorrelation, and recovery rate, can provide a dynamic signature of an impending thermally induced population collapse. By linking trait performance and climate research with knowledge of how demographic parameters generally respond to warming, early warning frameworks may ultimately prove to be a useful diagnostic tool in predicting temperature‐driven shifts in ecosystem structure and function.

## Conflict of Interest

None declared.

## Supporting information


**Figure S1.** Three possible temperature responses of maximum growth rate (*r*) and carrying capacity (*K*).
**Table S1.** AIC scores for *r‐* and *K‐* temperature models.
