# Supplementary material for: Early warning signals detect critical impacts of experimental warming
Source: Ecol Evol. 2016 Jul 29;6(17):6097–106. doi: 10.1002/ece3.2339 (PMC5016634; doi:10.1002/ece3.2339)
Supplement: Supplementary file 1 — Figure S1. Three possible temperature responses of maximum growth rate (r) and carrying capacity (K). Table S1. AIC scores for r‐ and K‐ temperature models. [file ECE3-6-6097-s001.pdf]

SUPPLEMENTARY INFORMATION

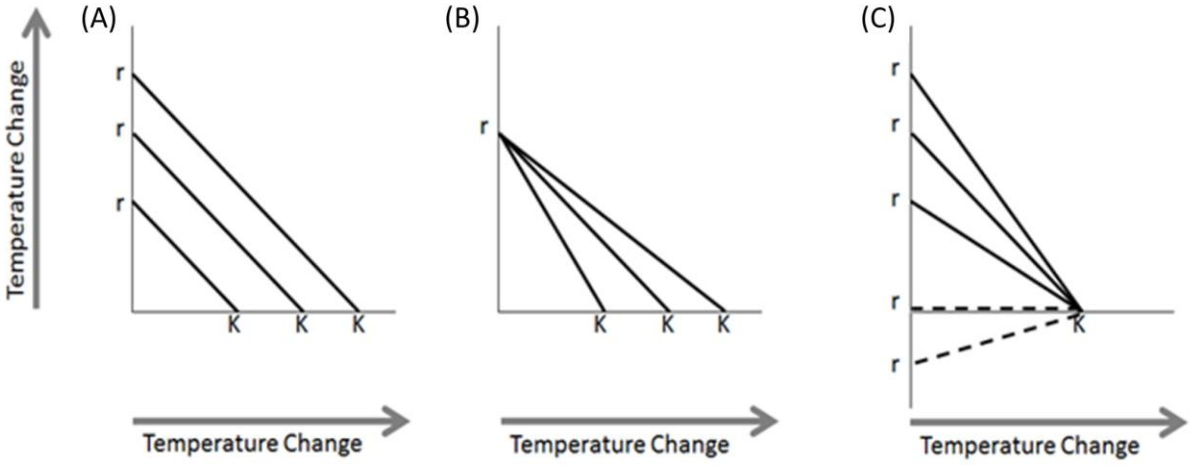

Fig. S1: Three possible temperature responses of maximum growth rate (*r*) and carrying capacity (*K*). The three possible scenarios describing the responses of maximum growth rate (*r*) and carrying capacity (*K*) with changes to temperature. Each line represents growth rate (r_t) regressed against abundance (N_t) at a different temperature. Plot (A) is showing a case where *r* and *K* are both temperature-dependent, (B) is a case where only *K* is temperature-dependent, and (C) is showing a case, similar to that shown in our study, where only *r* is temperature-dependent. For the third case (C), *K* is undefined for negative values of *r* and therefore is not biologically relevant and cannot be estimated.

Table 1: AIC scores for *r*- and *K*- temperature models.

Parameter	Model	AIC	Δ_{AIC}
r	**Logarithmic**	**-50.6**	**-2.3**
	**Exponential**	**-42.1**	**-10.8**
	**Quadratic**	**-52.9***	**0**
	**Linear**	**-24.9**	**-30.0**
	**Null**	**-18.8**	**-34.1**
K	**Logarithmic**	**281.9**	**-2.0**
	**Exponential**	**281.9**	**-2.0**
	**Quadratic**	**281.9**	**-2.0**
	**Linear**	**281.5**	**-1.6**
	**Null**	**279.9***	**0**

AIC scores for the models used to determine the thermal performance curves of the parameters r and K. ΔAIC=AICmin- AIC_i, where *i* is the alternate model. *most parsimonious model.
